# Impact of implementing a vancomycin protocol to reduce kidney toxicity: A comparative study

**DOI:** 10.3389/fphar.2023.1154573

**Published:** 2023-09-28

**Authors:** Graziella Gasparotto Baiocco, Stephanie Greiner, Mário Borges Rosa, Cecília Dias Flores, Helena M. T. Barros

**Affiliations:** ^1^ Programa de Pós-Graduação em Ciências da Saúde, Universidade Federal de Ciências da Saúde de Porto Alegre, Porto Alegre, Brazil; ^2^ Gestão de Risco Assistencial do Hospital Nossa Senhora da Conceição, Porto Alegre, Brazil; ^3^ Presidente do Instituto para Práticas Seguras no Uso de Medicamentos, ISMP, Belo Horizonte, Brazil

**Keywords:** vancomycin, nephrotoxicity, acute kidney injury, patient safety, protocol, public health

## Abstract

**Introduction:** Vancomycin is a frequently used antibiotic for treating severe infections caused by multidrug-resistant, Gram-positive pathogens. To ensure its effectiveness and minimize the risk of nephrotoxicity, safe administration and dose monitoring are crucial. Understanding the impact of vancomycin serum levels on clinical outcomes is of paramount importance, necessitating improved knowledge on its use, dose monitoring, nephrotoxicity, and safe administration.

**Objective:** This study aimed to evaluate the incidence of acute kidney injury (AKI) in patients receiving vancomycin before and after the implementation of an institutional protocol for vancomycin administration in a public tertiary hospital in southern Brazil.

**Materials and methods:** A cross-sectional study design was employed, analyzing data from the electronic medical records of 422 patients who received vancomycin. The patient population was divided into two independent cohorts: those treated in 2016 (pre-protocol) and those treated in 2018 (post-protocol), following the implementation of the institutional vancomycin administration protocol.

**Results:** The study included 211 patients in each year of assessment. Patients from both cohorts had a Charlson Comorbidity Index (CCI) score of 4. The post-protocol cohort consisted of older individuals, with a mean age of 62.8 years. In addition, patients in the post-protocol year had higher baseline creatinine levels, higher rates of intensive care unit (ICU) hospitalization, and increased use of vasopressors. In the pre-protocol year, patients received vancomycin therapy for a longer duration. When comparing the incidence of AKI between the two groups, an intervention study revealed rates of 38.4% in group 1 and 20.9% in group 2, indicating a significant reduction (*p* < 0.001) in the post-protocol group. A logistic regression model was developed to predict AKI, incorporating variables that demonstrated significance (*p* ≤ 0.250) in bivariate analysis and those recognized in the literature as important factors for AKI, such as the duration of therapy, vancomycin serum level, and ICU hospitalization. The logistic regression classification performance was assessed using a receiver operating characteristic (ROC) curve, yielding an area under the curve of 0.764, signifying acceptable discrimination of the regression model.

**Conclusion:** Implementation of the institutional protocol for vancomycin administration resulted in a significant and cost-effective impact, ensuring appropriate therapeutic dosing, reducing adverse events (e.g., nephrotoxicity), and improving clinical outcomes for patients in the study population.

## Introduction

The widespread use of vancomycin in clinical practice as the preferred glycopeptide antibiotic for treating infections caused by Gram-positive bacteria, particularly methicillin-resistant *Staphylococcus aureus* (MRSA), increases ongoing concerns among healthcare professionals regarding its efficacy and safety (Zamoner et al., 2019). To ensure therapeutic effectiveness, minimize toxicity, and combat antibiotic resistance, individualized dosing and monitoring of vancomycin serum levels are essential (Zamoner et al., 2020). Proper prescription practices aim to achieve the desired therapeutic effect while avoiding nephrotoxicity and reducing the risks of acute kidney injury (AKI) and associated short- and long-term complications (Zamoner et al., 2020). Maintaining vancomycin serum concentration within the range of 15–20 μg/mL is crucial for effective treatment (Rybak et al., 2009; Rybak et al., 2009; Rybak et al., 2020).

According to the 2020 consensus guidelines for therapeutic monitoring of vancomycin in adult patients, the optimal approach for dosing management involves assessing the area under the concentration–time curve (AUC) of vancomycin during the course of therapy (Rybak et al., 2020). Understanding the exposure-effect and exposure-toxicity relationships of the drug is critical in determining the pharmacodynamic index for vancomycin use, with an AUC/MIC ratio of ≥400 required for achieving bactericidal activity against MRSA. Despite this consensus, several hospitals still rely on monitoring trough serum concentrations of vancomycin due to challenges in implementing the Bayesian modeling techniques necessary for determining AUC/MIC.

Serum trough concentrations of 15–20 μg/mL are commonly used as a surrogate marker for achieving the optimal vancomycin AUC/MIC. Higher trough concentrations (>20 μg/mL) have been associated with an increased incidence of nephrotoxicity, with a greater risk observed at higher drug concentrations (Gyamlani et al., 2019; Rybak et al., 2020). Patients experiencing acute worsening of kidney function while using vancomycin face a higher risk of hospital mortality, more frequent requirements for hemodialysis, and longer hospital stays, resulting in increased healthcare costs (Zamoner et al., 2020).

The general objective of this study was to assess the incidence of AKI in patients using vancomycin before and after the implementation of an institutional protocol for vancomycin therapeutic drug monitoring at a general hospital in Brazil. The specific objective was to compare safety outcomes between the years before and after the protocol’s implementation. Understanding the impact of implementing an institutional protocol for safe vancomycin use can facilitate clinical decision-making and enhance the prescription and monitoring of vancomycin levels, with a focus on mitigating risks associated with its use.

## Materials and methods

### Study design

This study utilized a cross-sectional design to analyze data from electronic medical records of 422 patients who received vancomycin treatment. The patients were categorized into two independent groups, representing the periods before and after the implementation of the institutional protocol for vancomycin therapeutic drug monitoring.

### Setting

The study was conducted in a large general hospital in the city of Porto Alegre, Rio Grande do Sul, Brazil. The hospital provides care for adult clinical and surgical patients.

### Period

The institutional protocol development occurred between 2016 and 2017, with implementation taking place in 2017. Data for this study were collected retrospectively from patient charts covering 2016 (pre-protocol implementation) and 2018 (post-protocol implementation). The data collection process occurred in 2021.

### Population and sample

The list of all patients who received vancomycin prescriptions in 2016 and 2018 was obtained from the information technology department. The inclusion and exclusion criteria were met, and patient charts were randomly selected for data collection. The sample size calculation was performed using the Power and Sample Size for Health Researchers (PSS Health) software, version 2021 (Borges et al., 2021). A sample size of 422 patients categorized into two independent groups was determined, considering a power of 90% and a significance level of 1%. The objective was to detect differences between patients before and after the implementation of the protocol using the propensity score for nephrotoxicity. The proportion of nephrotoxicity before the protocol’s implementation was estimated at 39%, based on hospital statistics from 2016 (Helena Dias Pereira Dos Santos Ulbrich et al., 2022). The risk of nephrotoxicity in a Brazilian hospital with a monitoring protocol in place was estimated at 19% (De Almeida et al., 2019).

### Inclusion criteria

**FIGURE 1:**
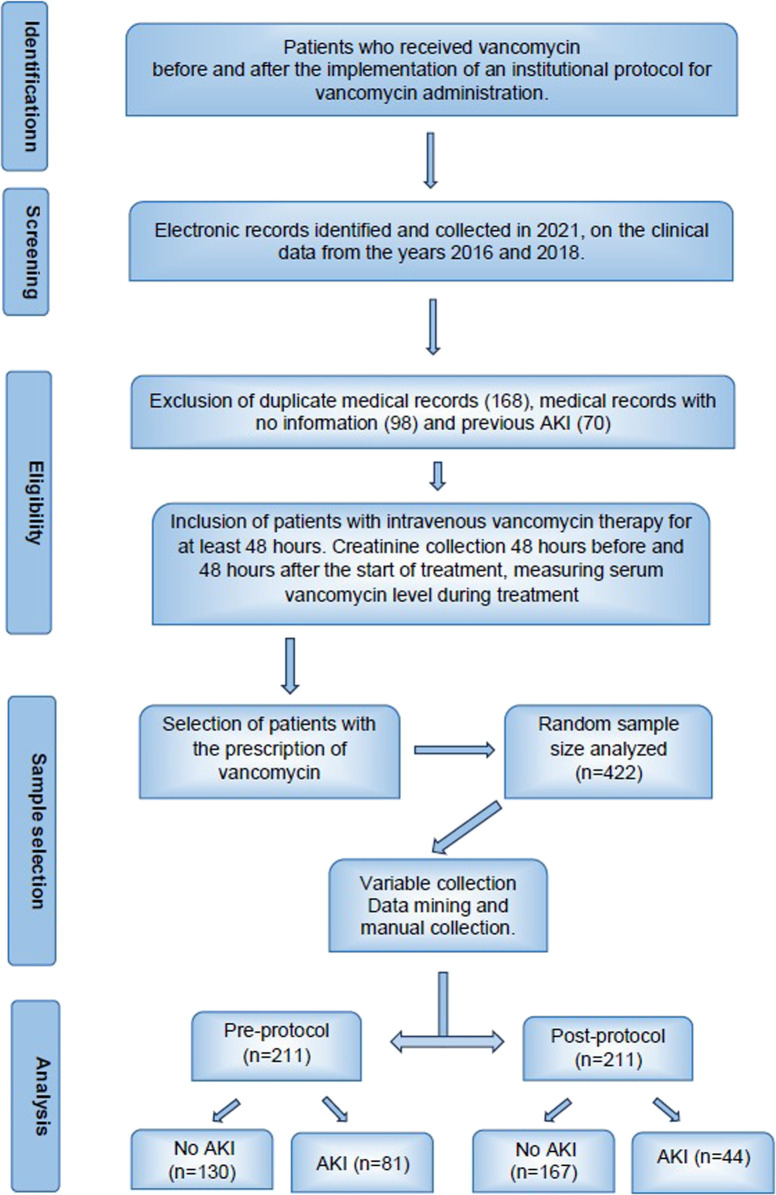
Flowchart of patient charts’ inclusion criteria.

The medical records of adult patients who received intravenous vancomycin therapy for at least 48 h were included. Patients with a creatinine collection 48 h before and 48 h after the start of treatment and a measurement of serum vancomycin concentration during treatment, dosed 1 hour before the third or fourth vancomycin dose, were also included (Figure 1). Patients with kidney function insufficiency (acute or chronic) up to 48 h before vancomycin use were excluded from the analysis. Data were extracted from electronic medical records through data mining, which identifies consistent patterns and associations between variables, as well as new subsets of data. Data not obtained automatically were manually collected by trained research workers. The included variables were vancomycin prescription days, maximum daily antibiotic dose, serum creatinine levels before and 48 h after vancomycin infusion, vancomycin serum level, weight, CCI determined at patient admission, gender, ethnicity, age, use of noradrenaline, use of nephrotoxic drugs, and mortality during hospitalization. The CCI score (Charlson et al., 1987), which consists of 20 variables associated with 10-year mortality, served as an indicator of disease burden. Other variables of interest included AKI (assessed through creatinine measurement during hospitalization), admission to the intensive care unit (ICU), and mortality during hospitalization. AKI was defined according to the classification of the Global Guidelines for Amelioration of Kidney Disease, which considers an increase in creatinine of ≥0.3 mg/dL within 48 h after the use of vancomycin as indicative of AKI (Khwaja, 2012).

Protocol for vancomycin administration: The objective of the protocol was to establish guidelines for the safe use of vancomycin, defining clinical indications and providing instructions for proper monitoring. The development and implementation of the institutional protocol for safe vancomycin administration were accomplished through a focus group methodology. This working group comprised professionals from various disciplines, including risk management and patient safety, the hospital infection control commission (HICC), prescribing physicians, clinical pharmacists, biochemists from the clinical analysis laboratory, and informatics management. The multidisciplinary rounds facilitated the formulation and implementation of the institutional protocol in 2017. The protocol’s creation involved an initial review of scientific evidence on the subject, revising the criteria for vancomycin use, establishing standard dose administration times, defining the optimal time for vancomycin serum level determination (critical for appropriate control), dose adjustments based on kidney function, determining the critical result value, and developing communication procedures for critical results to alert prescribers. The protocol was then designed, support teams were trained, and the protocol was disseminated institution-wide.

The protocol established that vancomycin would be prescribed to patients based on the HICC guidelines, with an initial dose of 12–20 mg/kg administered every 12 h (maximum 2 g/dose). Dose adjustments were specified for severe infections, obesity, and kidney or hemodialysis conditions. The protocol also determined that the first blood sample for determining the vancomycin serum level (VSL) should be collected less than 60 min before the third or fourth dose. It also outlined dosing adjustments based on VSL results. To validate the proposed protocol, discussions were held with inpatient clinical and surgical units, as well as ICU specialists, conducting several rounds of feedback among the professionals involved. Notably, the study institution did not have a previous vancomycin administration protocol in place.

Following these steps, training began in early 2017 and was continued throughout the year. The theoretical and practical training was provided to relevant areas of the institution, focusing on prescribing vancomycin, adjusting serum level collection times, communicating critical results, and adapting antibiotic dosage accordingly.

### Data analysis

The statistical treatment of the data was performed with the help of the Statistical Package for Social Sciences software, version 25.0. For statistical decision criteria, a significance level of 0.05 was adopted. The study of the symmetry of continuous distributions was analyzed by the Kolmogorov–Smirnov test. The treatment of missing data (maximum of 5.2% of the information) was performed by direct imputation (mean or median scores), depending on the symmetry of data distribution. For the bivariate analysis between categorical variables, Pearson’s chi-squared test (χ2) was used, with the estimation of the measure of effect performed by the crude odds ratio (OR). For continuous variables, Student’s *t*-test or the Mann–Whitney *U* test was used for the comparison between two independent groups. The comparison of continuous variables between more than two independent groups was carried out through the analysis of variance for two factors (ANOVA two-way)—*post hoc* Bonferroni. In order to identify the independent variables with predictive/explanatory capacity for AKI, the multivariate binary logistic regression technique was used. The representative variables in the model were listed by the backward conditional method from the saturated model. The association was assessed with the likelihood ratio test -2log, and the Nagelkerke and Hosmer–Lemeshow R^2^ estimators were considered to assess the goodness of fit. The probability of gradual entry of the variables to the model was 0.05, and for removal, it was 0.10. Over the cut-off point, the significance was 0.50 for a maximum of 20 interactions. Furthermore, the estimation of the ROC was considered to assess the predictive capacity of the final regression model (Zweig and Campbell, 1993; Hosmer and Lemeshow, 2000; Hair et al., 2009).

### Ethical aspects

The study is part of an umbrella project, which was approved through Plataforma Brasil, Research Ethics Committee of the Institution under the number 3.911.811. Patient data were kept confidential and accessed only by research members.

## Results

The presented results pertain to the comparison of data from patients categorized based on the time of their hospitalization, that is, before and after the implementation of the institutional protocol for safe vancomycin administration. Table 1 provides a comprehensive characterization of the sample, including sociodemographic data, divided into pre-protocol (2016) and post-protocol (2018) groups, stratified according to severity markers and outcome markers.

**TABLE 1:** Sociodemographic and clinical characteristics of study participants by the year of assessment.

Characterization of the sample	Year and AKI^A^		*p*
	Pre-protocol (*n* = 211)	Post-protocol (*n* = 211)	
Female gender, n (%)	125 (59.2)	129 (61.1)	0.246^d^
White race^c^, n (%)	172 (81.5)	160 (76.2)	0.096^e^
Age (years)	59.0 ± 17.3	62.8 ± 15.4	0.018^g^
Severity markers			
Previous creatinine (mg/dL)^b^	0.879 (0.64–1.17)	1.14 (0.76–1.84)	<0.001^f^
Hospitalized in the ICU (yes), n (%)	62 (29.4)	87 (41.2)	0.011^d^
Vasopressors use, n (%)	48 (22.7)	78 (37.0)	0.001^d^
Weight (kg)^c^	67.9 ± 16.8	68.7 ± 18.2	0.328^g^
CCI^b^	4.0 (3.0–6.0)	4.0 (2.0–5.0)	0.253^f^
CCI >3 (number), n (%)	123 (58.3)	131 (62.1)	0.426
Nephrotoxic drugs prescribed, n (%)	211 (100.0)	208 (98.6)	0.073^e^
Outcome markers			
Days of vancomycin therapy (number)^b^	8 (6–12)	6 (4–9)	<0.001^f^
Acute kidney injury (yes), n (%)	81 (38.4)	44 (20.9)	<0.001^d^
Vancomycin serum level (mcg/mL)^b^	24.5 (16.80–33.60)	26.95 (20.70–32.60)	0.119^f^
Outcome (death), n (%)	69 (32.7)	79 (37.4)	0.308^d^

^a^ Percentages obtained based on the total number of valid cases and each year.

^b^ Asymmetric distribution (Kolmogorov–Smirnov; *p* < 0.05), estimates presented by [median (1st–3rd quartile)].

^c^ Missing data: ethnicity [2018—1 (0.5%)].

^d^ Pearson’s chi-squared test.

^e^ Fisher’s exact test.

^f^ Mann–Whitney *U* test.

^g^ Student’s *t*-test for independent groups.

In both pre- and post-protocol years, most patients were of white ethnicity, with more than 50% being females. Patients in both groups exhibited similar body weight, and upon arrival at the hospital, they had an identical mean CCI severity score of 4. However, the post-protocol group of patients was significantly older, with the mean age being 3 years higher than that of the pre-protocol group. Notably, the creatinine levels prior to vancomycin use were significantly higher in the post-protocol year, and this group also had a higher incidence of being admitted to the ICU and being prescribed vasopressors, indicating that they were in more severe clinical conditions at the time of vancomycin prescription. In contrast, patients in the pre-protocol year received vancomycin therapy for 25% more days compared to those in the post-protocol year.

The most striking finding is that significantly fewer patients (50% less) developed AKI in the post-protocol year (20.9%) than in the pre-protocol year (38.4%). However, there was no observed change in mortality between the two groups.

The main outcomes of the study were to verify the incidence of AKI and VSLs (≤20 μg/mL and >20 μg/mL), when comparing the pre- and post-protocol years. Intervening factors were the variables of the general profile of the patients in the samples, in each year of assessment. According to the results shown in Tables 2, 3, the factors related to AKI incidence in the periods were as follows: having higher pre-protocol vancomycin serum levels, serum level classification higher than 20 μg/mL, and more days of therapy with vancomycin. It was observed that age, sex, and being white did not vary in a representative way in relation to the pre- and post-protocol years and AKI levels.

**TABLE 2:** Mean, standard deviation, and median for age and clinical characteristics according to the year and AKI.

Characterization of the sample	Year and AKI				Effects		
	Pre-protocol (n = 211)		Post-protocol (n = 211)		Year	AKI	Interaction
	Non-AKI (*n* = 130)	AKI (*n* = 81)	Non-AKI (*n* = 167)	AKI (*n* = 44)			
Age (years)	58.8 ± 17.2	29.3 ± 17.7	63.1 ± 15.7	61.9 ± 14.7	0.060	0.867	0.676
Vancomycin serum level (μg/mL)	21.8 ± 10.0 (20.3)	33.3 ± 13.9 (32.5)	28.2 ± 13.7 (26.9)	29.6 ± 13.1 (26.9)	0.324	<0.001	<0.001
Days of therapy^J^	9.5 ± 5.9 (8.0)	10.8 ± 6.8 (9.0)	7.4 ± 5.6 (6.0)	8.6 ± 6.5 (6.0)	0.001	0.069	0.873
CCI scores ^J^	4.4 ± 2.5	4.6 ± 3.2	4.1 ± 2.5	3.9 ± 2.1			
Previous creatinine (mg/dL) ^J^	1.11 ± 0.76 (0.88)	0.99 ± 0.56 (0.90)	1.55 ± 1.84 (1.04)	2.18 ± 2.08 (1.71)	<0.001	0.103	0.018

Test effects, analysis of variance (2 factors)—*post hoc* Bonferroni; effects estimates for year, AKI, and year*IRA interaction; J, Results presented as mean ± standard deviation (median).

**TABLE 3:** Absolute and relative distribution of age and clinical characteristics according to the year and AKI.

Characterization of the sample	Year and AKI^A^			
	Pre-protocol (*n* = 211)		Post-protocol (*n* = 211)	
	Non-AKI (*n* = 130)	AKI (*n* = 81)	Non-AKI (*n* = 167)	AKI (*n* = 44)
Serum level—classification				
≤20 μg/mL, n (%)	64 (42.9)	15 (18.5)	41 (24.6)	7 (15.9)
>20 μg/mL, n (%)	66 (50.8)	66 (81.5)	126 (75.4)	37 (84.1)
p^D^	<0.001		0.224	
Use of vasopressor				
Not used, n (%)	112 (86.2)	51 (63.0)	113 (67.7)	20 (45.5)
Used, n (%)	18 (13.8)	30 (37.0)	54 (32.3)	24 (54.5)
p^D^	<0.001		0.007	
Outcome				
Hospital discharge, n (%)	99 (76.2)	43 (53.1)	112 (67.1)	20 (45.5)
Death, n (%)	31 (23.8)	38 (46.9)	55 (32.9)	24 (54.5)
p^D^	0.001		0.008	
CCI classification				
≤3, n (%)	51 (39.2)	37 (45.7)	59 (35.3)	21 (47.7)
>3, n (%)	79 (60.8)	44 (54.3)	108 (64.7)	23 (52.3)
p^D^	0.356		0.132	
Hospitalization in the ICU				
Was not hospitalized in the ICU, n (%)	109 (83.8)	40 (49.4)	103 (61.7)	21 (47.7)
Was hospitalized in the ICU, n (%)	21 (16.2)	41 (50.6)	64 (38.3)	23 (52.3)
_p_D	<0.001		0.094	

A, percentages obtained based on the total number of valid cases and each year; D, Pearson’s chi-squared test.

Regarding the severity markers of clinical conditions and the use of noradrenaline (vasopressor), the results demonstrated a significant association between the absence of AKI and non-use of vasopressors. Conversely, patients who experienced AKI were associated with the use of vasopressors.

Hospital discharge was significantly associated to non-AKI patients, as 76.2% of individuals with this outcome were not affected by AKI. Conversely, in cases where the outcome was death, 46.9% of patients were associated with AKI. These associations were consistent in both pre- and post-protocol years.

Furthermore, hospitalization in the ICU exhibited a significant association with AKI (p < 0.001) in the pre-protocol year. In this period, 50.6% (*n* = 41) of patients who experienced AKI were also hospitalized in the ICU. Similarly, in the post-protocol year, 52.3% (*n* = 23) of patients who developed AKI were admitted to the ICU.

In Tables 4, 5, the results presented are related to the comparisons of the variables of the patients’ profile with the classification for the VSL (≤20 μg/mL and >20 μg/mL), pre- and post-protocol years; it was found that for age, a significant effect was detected [F (1; 418) = 6.092; *p* = 0.014] and for the serum level [F (1; 418) = 6.349; *p* = 0.012]. Our results indicate that the mean age in the post-protocol year was significantly higher than that in the pre-protocol year (regardless of the serum level). As for the representative effect on the classifications for the serum level, cases of patients with a serum level >20 μg/mL have a higher mean age than cases of patients with a serum level ≤20 μg/mL. Therefore, the older the patients, the higher the concentration of the serum level.

**TABLE 4:** Mean, standard deviation, and median for age and clinical characteristics according to the year and serum level.

Characterization of the sample	Year and serum level (μg/mL)				Effects		
	Pre-protocol (*n* = 211)		Post-protocol (*n* = 211)		Year	AKI	Interaction
	Serum level ≤ 20 (*n* = 79)	Serum level > 20 (*n* = 132)	Serum level ≤ 20 (*n* = 48)	Serum level > 20 (*n* = 163)			
Age (years)	54.3 ± 16.0	61.8 ± 17.3	61.7 ± 16.2	63.1 ± 15.2	0.014	0.012	0.083
Days of therapy^J^	9.3 ± 5.5 (8.0)	10.4 ± 6.8 (8.0)	7.8 ± 5.0 (7.0)	7.7 ± 5.8 (6.0)	0.001	0.510	0.318
CCI (scores) ^J^	4.3 ± 2.6 (4.0)	4.6 ± 2.9 (4.0)	3.6 ± 2.1 (4.0)	4.2 ± 2.5 (4.0)	0.065	0.130	0.630
Previous creatinine (mg/dL) ^J^	0.92 ± 0.54 (0.77)	1.15 ± 0.76 (0.94)	1.07 ± 0.82 (0.88)	1.85 ± 2.09 (1.25)	0.005	0.001	0.071

Effects, analysis of variance (2 factors)—*post hoc* Bonferroni; Effect estimates for year, serum level, and interaction year*serum level; J, results presented as mean ± standard deviation (median).

**TABLE 5:** Absolute and relative distribution of clinical characteristics by the serum level.

Characterization of the sample	Year and serum level (μg/mL)			
	Pre-protocol (*n* = 211)	Post-protocol (*n* = 211)	Pre-protocol (*n* = 211)	Post-protocol (*n* = 211)
	Serum level ≤20 (*n* = 79)	Serum level >20 (*n* = 132)	Serum level ≤20 (*n* = 79)	Serum level >20 (*n* = 132)
Use of vasopressor				
Did not use, n (%)	72 (91.1)	91 (68.9)	36 (75.0)	97 (59.5)
Used, n (%)	7 (8.9)	41 (31.1)	12 (25.0)	66 (40.5)
_p_D	<0.001		0.051	
Outcome				
Hospital discharge, n (%)	61 (77.2)	81 (61.4)	42 (87.5)	90 (55.2)
Death, n (%)	18 (22.8)	51 (38.6)	6 (12.5)	73 (44.8)
_p_D	0.018		<0.001	
CCI classification				
≤3 points, n (%)	35 (44.3)	53 (40.2)	20 (41.7)	60 (36.8)
>3 points, n (%)	44 (55.7)	79 (59.8)	28 (58.3)	103 (63.2)
_p_D	0.554		0.543	
Hospitalization in the ICU				
Was not hospitalized in the ICU, n (%)	65 (82.3)	84 (63.6)	33 (68.8)	91 (55.8)
Was hospitalized in the ICU, n (%)	14 (17.7)	48 (36.4)	15 (31.2)	72 (44.2)
_p_D	0.004		0.110	

A, percentages obtained based on the total number of valid cases of each severity classification and in each year; D, Pearson’s chi-squared test.

Regarding the use of noradrenaline (vasopressor), a significant association was observed when comparing the serum level classification in each year. In the pre-protocol year (*p* < 0.001), patients with serum levels ≤20 μg/mL were significantly associated with not using vasopressors (91.1%, *n* = 72). Conversely, patients with serum levels >20 μg/mL were significantly associated with the use of vasopressors (31.1%, *n* = 41). However, in the post-protocol year, a significant association was not found. This suggests that in the post-protocol year, the use of vasopressors was prevalent in most patients whose serum levels fell outside the appropriate range.

The characterization of the outcome was significantly associated with the serum level in both years of assessment. In the pre-protocol year (*p* = 0.018), patients discharged from the hospital were significantly associated with serum levels ≤20 μg/mL (77.2%, *n* = 61), whereas cases resulting in death were significantly associated with serum levels >20 μg/mL (38.6%, *n* = 51). Similar results were found in the post-protocol year (*p* < 0.001), highlighting the influence of serum levels within the normal range on adequate recovery and favorable outcomes such as hospital discharge.

Regarding the number of days of prescribed vancomycin therapy, compared to the serum level and the year, a significant effect was detected exclusively in the pre-protocol assessment year [F (1; 418) = 10.472, *p* < 0.001]. The pre-protocol year saw a higher concentration of prescribed therapy days than the post-protocol year.

Significant effects were also observed for the previous creatinine levels, with significant differences between years [F (1; 418) = 7.852, *p* = 0.005] and serum levels [F (1; 418) = 10.956, *p* = 0.001]. The mean pre-protocol creatinine level in the post-protocol year was significantly higher than that in the pre-protocol year (regardless of the serum level). These data reflect patients’ previous creatinine values, which were the clinical data before prescribed therapy, and could not be modified. In addition, patients with a serum level >20 μg/mL had higher mean levels of previous creatinine than patients with a serum level ≤20 μg/mL, indicating the influence of patients’ kidney function on the VSL in the blood.

Being hospitalized in the ICU showed a significant association with AKI (p = 0.004) only in the pre-protocol year. In this period, patients with a serum level ≤20 μg/mL were significantly associated with not being hospitalized in the ICU (82.3%, *n* = 65), whereas cases of patients with serum levels >20 μg/mL were significantly associated with hospitalization in the ICU (36.4%, *n* = 48). However, in the post-protocol year, these variations were not statistically significant.

**FIGURE 2:**
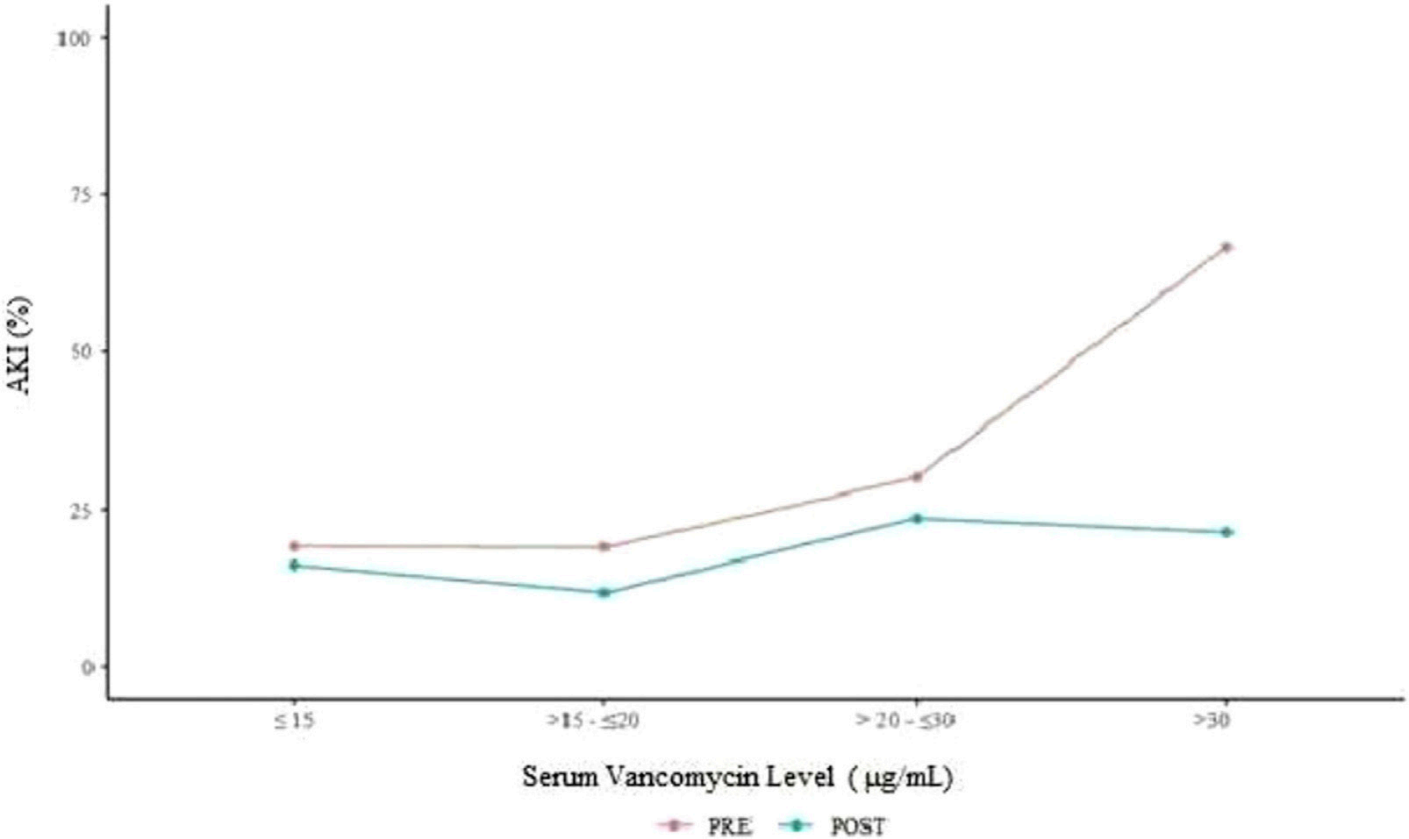
AKI incidence in relation to the VSL.

When stratifying patients by the VSL as <15, 15–20, 20–29, and >30 μg/mL, the incidence of AKI was higher in the pre-protocol year; however, this difference was statistically significant when comparing patients with VSL >30 μg/mL (*p* < 0.001) (Figure 2). Considering the association between AKI and the serum level, in the same year, a statistically significant association was detected (*p* < 0.001), which did not occur in the post-protocol year (*p* = 0.632). In the data referring to the post-protocol year, the different ranges for the serum level were not related to AKI.

Based on Figure 2, it is evident that in the pre-protocol year, a considerable number of patients had VSLs higher than 30 μg/mL, indicating an increased risk of AKI. The differences in the proportions of patients who experienced AKI in the pre- and post-protocol assessments were particularly pronounced in the classification for a serum level greater than 30 μg/mL. To better understand this result, risk estimates (gross odds ratio, gOR) were computed. In the pre-protocol assessment, the risk for AKI in patients with a serum level greater than 30 μg/mL was 8.5 times higher (CI 95% = 3.412–21.177) than that in patients with a serum level ≤15. However, in the post-protocol assessment, this estimate was not statistically significant (gOR = 1.408; 95%CI: 0.452–4.388).

The initial multivariate logistic regression model included variables that, in the bivariate analysis (when compared to AKI), exhibited a significance level of *p* ≤ 0.250, as well as those known in the literature to be crucial factors for AKI. The previous creatinine variables and the CCI score were not included in the model, despite their significance, as the objective was to study the variables controlled by the study protocol. The research workers acknowledged the relevance of patients’ previous characteristics before starting vancomycin therapy in determining the risk of AKI.

As shown in Table 6, the ideal model was established in four steps, resulting in the exclusion of four variables that did not significantly contribute as other variables (use of vasopressor, age, race, and gender). Among the factors considered representative in the final model, the year of patients’ enrollment had the greatest impact, with patients in the pre-protocol year having odds ratio (OR) 3.641 [95%CI: 2.238–5.927] times higher likelihood of experiencing AKI than patients in the post-protocol year. The second factor with a significant impact on AKI occurrence was hospitalization in the ICU, where patients treated in this unit had OR: 2.673 [95%CI: 1.621–4.406] times higher likelihood of developing AKI than patients not in the ICU. The VSL also remained significant in the model, indicating that higher VSL was associated with a greater chance of experiencing AKI, with OR: 1.254 [95%CI: 1.228–1.334], than those with lower levels. In addition, the days of prescribed therapy with vancomycin were also found to be significant, with patients on longer therapy being more likely to develop AKI, with OR: 1.266 [95%CI: 1.226–1.319].

**TABLE 6:** Binary logistic regression models to analyze the incidence of AKI.

Independent variable	Model for analyzing the incidence of AKI			
	Odds ratio	IC95% odds ratio		*p*
		Lower	Higher	
*Early model*				
Year (pre-protocol)	3.581	2.008	6.386	<0.001
Days of therapy	1.225	1.181	1.270	0.109
Age	1.179	1.162	1.196	0.287
Vancomycin serum level	1.228	1.206	1.250	<0.001
Hospitalization in the ICU (yes)	2.424	1.184	4.962	0.038
Vasopressor use (yes)	1.334	0.615	2.899	0.814
Gender (female)	1.083	0.521	2.251	0.714
White race	2.405	1.413	4.094	0,006
Constant	0.056			<0.001
*Final model*				
Year (pre-protocol)	3.64	2.238	5.927	<0.001
Days of therapy	1.26	1.226	1.319	0.026
Vancomycin serum level	1.25	1.228	1.334	0.009
Hospitalization in the ICU (yes)	2.67	1.621	4.406	<0.001
Constant	0.03			<0.001

Initial model—regression model parameters: Nalgelkerke’s R^2^ = 0.316; Cox & Nel = 0.202; 2LL, 445.123; Hosmer–Lemeshow test—Chi square (8) = 11.451; *p* = 0.256; confusion matrix: total 76.2%. Final model—regression model parameters: Nalgelkerke’s R^2^ = 0.336; Cox & Nel = 0.224; 2LL, 442.546; Hosmer–Lemeshow test—Chi square (8) = 16.564; *p* = 0.178; confusion matrix: total 79.97%.

**FIGURE 3:**
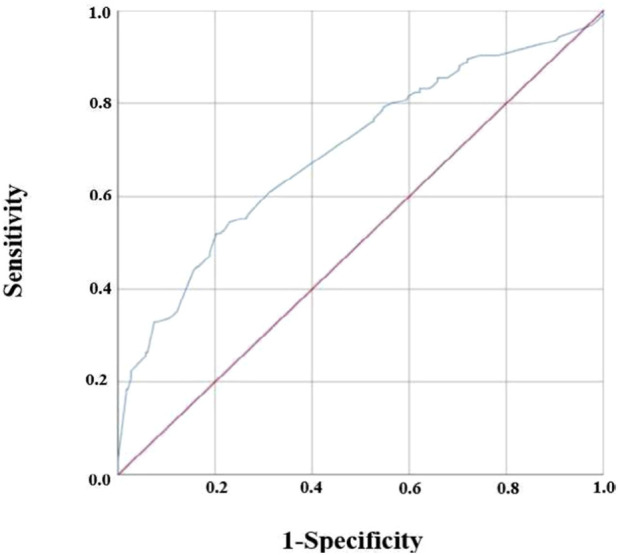
ROC of the regression model.

The classification performance of this logistic regression was investigated through the ROC curve, showing how well the model created distinguished between individuals who had AKI and those who did not have AKI, as shown in Figure 3.

The prediction estimation for the probability of high VSL, longer therapy duration, and ICU hospitalization associated with AKI is 0.764 (*p* < 0.001).

Sensitivity refers to the accurate classification of AKI, meaning that in the actual sample, the patient indeed had AKI. Specificity, on the other hand, refers to the accurate classification of no AKI, indicating that, in the real sample, the patient did not have AKI.

## Discussion

The objectives of this study were twofold: first, to compare the incidence of AKI in hospitalized patients receiving vancomycin before and after the implementation of an institutional protocol for vancomycin administration, and second, to analyze the contributing factors related to nephrotoxicity risks.

In the pre-protocol year, vancomycin prescription occurred with/without adequate dosing and consideration of previous creatinine levels to determine the dose or interval. The implemented protocol specified that vancomycin would be prescribed to patients at a dose of 12–20 mg/kg, not exceeding 2 g per day, with a 12-h interval. Moreover, the protocol dictated that blood samples for VSL determination should be collected less than 60 min before the third or fourth dose, and subsequent dose and frequency adjustments should be made according to the recommendations of the protocol. This study demonstrates that the number of days of therapy with vancomycin was reduced after the protocol implementation, potentially contributing to a lower incidence of AKI, shorter hospitalization stays, fewer ICU admissions, and lower mortality rates. These positive outcomes were observed despite the fact that the study population in the post-protocol year consisted of older patients with higher creatinine levels before initiating vancomycin treatment.

Corroborating with the study’s findings, in the pre-protocol year, patients who experienced AKI had significantly higher mean VSLs than those who did not have AKI. However, in the post-protocol year, the difference between the means of VSLs was not substantial. Analyzing the VSLs based on their classification (≤20 and >20 μg/mL), it was observed that in the pre-protocol year, patients with AKI were significantly associated (*p* < 0.001) with the >20 μg/mL range (81.5%, *n* = 66). Conversely, patients without AKI were associated with ≤20 μg/mL levels (42.9%, *n* = 64). In the post-protocol year, no significant results were observed (Van Hal et al., 2013).

Although vancomycin is widely used in the treatment of severe infections and its efficacy is supported by various published studies, it is of great relevance to quantify the risk and factors leading to the occurrence of complications related to its use, especially in situations where the patient’s health status is critical (Al-Sulaiti et al., 2019).

Patients who are critically ill may present physiological changes that can interfere with the pharmacokinetic properties of antimicrobials, potentially leading to alterations in the apparent volume of distribution, plasma clearance, and reduction of biological half-life, which affects the optimization of the vancomycin dosing regimen. In this sense, vancomycin monitoring is extremely necessary for pharmacotherapy optimization, in order to minimize subtherapeutic or toxic concentrations (Alves et al., 2019).

Monitoring VSLs and appropriately adjusting the dose are of utmost importance to limit toxicity. The implementation of a vancomycin control protocol, along with educational and training measures, plays a crucial role in providing guidance and dissemination of resources to enhance vancomycin dosing and minimize toxicity. A systematic review (Phillips et al., 2017) indicated that proper guidance and monitoring of vancomycin can significantly reduce the chances of adverse events, making it an effective antibiotic despite being in use for over half a century.

Individualizing the dosage and conducting frequent reassessments are essential for optimizing drug efficacy, minimizing toxicity, and addressing resistance emergence. The multidisciplinary team should regularly reevaluate the rationale for continuing vancomycin therapy and discontinue it if no longer indicated. Ongoing studies and active debates continue to explore the optimal approach in monitoring vancomycin serum concentration for managing invasive MRSA infections (Jorgensen et al., 2021; Lodise and Drusano, 2021).

For the treatment of serious MRSA infections, it is recommended to achieve a pharmacokinetics and pharmacodynamics target resulting in an AUC and minimum inhibitory concentration (MIC) greater than 400 after 24 h (Rybak et al., 2009). Due to the inherent challenges in obtaining AUC/MIC, the Infectious Diseases Society of America (ISDA), the American Society of Health-System Pharmacists, and the Infectious Diseases Society of Pharmacists suggest monitoring vancomycin trough plasma levels as a practical alternative, given its good correlation with AUC/MIC. The recommended range for vancomycin trough plasma levels is between 15 and 20 μg/mL, with levels ≤10 μg/mL be avoided. The plasma level should be measured within 30 min before the infusion of the fourth or fifth dose of the antibiotic, following the initial dose or adjustment (Liu et al., 2011). A systematic review and meta-analysis supported the adherence to these therapeutic levels, showing improved clinical outcomes and reduced nephrotoxicity (Ye et al., 2014), which align with the findings of our study and the implemented institutional protocol.

Prolonged administration of vancomycin has been associated with a substantial increase in nephrotoxicity (Rezende et al., 2021). Our study, involving critically ill adult patients, revealed that nephrotoxicity occurred between 4 and 17 days after initiating vancomycin therapy, reinforcing the importance of reduced therapy duration after protocol implementation and the subsequent reduction of nephrotoxicity.

A cohort study conducted by Qin et al. (2020) with over 12,700 patients from Hong Kong highlighted the significance of monitoring VSLs in patients with previous baseline creatinine above normal and concomitant use of nephrotoxic drugs, particularly diuretics, piperacillin–tazobactam, and meropenem. Our study also identified almost 100% of patients using nephrotoxic drugs concurrently with prescribed vancomycin therapy, emphasizing the importance of careful monitoring in such cases.

The data supporting the use of dose adjustment based on trough serum concentration to optimize medication efficacy are scarce. In a systematic review that included over 2,000 patients with invasive MRSA infection, no difference in mortality was observed between patients with minimum concentrations of <15 mcg/mL *vs.* ≥15 mcg/mL (Steinmetz et al., 2015). Another study, a meta-analysis including over 1,600 patients with *S. aureus* bacteremia, did not observe any correlation between concentrations >15 mcg/mL and treatment failure rates, persistent bacteremia, or mortality (Prybylski, 2015).

Nephrotoxicity remains the most severe adverse effect associated with vancomycin, as reported by many studies, and is associated with increased mortality, hospital stay, and increased medical expenses (Jeffres, 2017). Risk factors for nephrotoxicity include the dose and duration of vancomycin treatment, serum concentration through serum level, patient characteristics, and continuous administration of nephrotoxic drugs (Van Hal et al., 2013; Jeffres, 2017).

In a retrospective study comparing the incidence of nephrotoxicity with AUC-guided dosing *vs.* trough monitoring in 1,280 vancomycin-treated patients, AUC-guided dosing was associated with a lower likelihood of AKI (Finch et al., 2017).

Another prospective study including 252 vancomycin-treated patients monitored with trough levels of 10–20 mg/L or estimated AUC values of ≥400 mg h/L by Bayesian methods showed a nephrotoxicity probability of 8% *vs.* 0–2%, respectively (Neely et al., 2018).

Among patients with AKI, the median vancomycin trough concentration was 15.7 mg/L and the median AUC was 625 mg h/L. Among patients without AKI, the median vancomycin trough concentration was 8.7 mg/L and the median AUC was 423 mg h/L.

Implementation challenges associated with AUC-based monitoring include the need for staff education, protocol development, consideration of software acquisition, and workflow integration for healthcare professionals, laboratory staff, and pharmacists. Despite these challenges, some hospitals in the United States have successfully adopted the AUC estimation approach.

In the logistic regression model, the selection of variables for the ideal model occurred in four steps. Out of all the initially included variables, four were excluded from the model as they did not significantly contribute as other variables (vasopressor use, age, race, and gender). The final representative model revealed two factors with the most significant impact on the occurrence of AKI. First, the year of investigation played a pivotal role, as patients from the pre-protocol year were 3.641 times more likely to experience AKI than those from the post-protocol year. This indicates that the implementation of the institutional protocol had a positive effect in reducing the risk of AKI in patients receiving vancomycin. Second, hospitalization in the ICU was also strongly associated with the occurrence of AKI, with patients treated in the ICU being 2.673 times more likely to develop AKI than patients not hospitalized in the ICU. Moreover, VSLs remained a significant factor in the model, indicating that higher levels of vancomycin were linked to an increased likelihood of developing AKI, as opposed to lower levels of the drug. Last, the number of days of prescribed therapy also demonstrated significance in the model, with patients who received longer vancomycin therapy being more prone to developing AKI.

The institutional protocol developed by the hospital’s multidisciplinary team in this study aimed to define indications and procedures for monitoring vancomycin levels in clinical practice. As the hospital does not have a program to estimate AUC values using Bayesian tools, the proposal was to use trough concentrations of vancomycin (Rybak et al., 2009; Rybak et al., 2020; Lodise and DrusanO, 2021). The objectives of the team were to establish guidelines, define indications, guide sample collections, and establish monitoring procedures for vancomycin levels. It is important to note that this protocol has been reviewed and is regularly updated every three years.

This study has several limitations. First, it is a single-center observational study, which may limit the generalizability of the findings to other settings. In addition, being an observational study, it does not allow for the control of confounding biases that could influence the results. For instance, the study includes both clinical patients hospitalized in wards and critically ill patients admitted to the ICU, making it challenging to isolate the specific impact of vancomycin on nephrotoxicity. Furthermore, there are several exclusion variables, such as the presence of AKI of other etiologies at the time of hospitalization in the ICU or the introduction of vancomycin, which might have affected the outcomes.

## Conclusion

In conclusion, this study highlights the positive impact of educational and low-cost institutional intervention focused on mitigating adverse drug-related events associated with vancomycin therapy. By implementing an institutional protocol for safe vancomycin administration, which includes appropriate dosing, timely monitoring of serum levels, and effective communication of critical results, early detection of toxic levels can be achieved. This, in turn, enables timely therapeutic interventions, such as dose adjustments or administration intervals, ultimately leading to a reduced risk of AKI and potentially lower mortality rates.

The management of vancomycin remains challenging due to several factors related to patient characteristics and drug pharmacokinetics, particularly in critically ill patients where severity of the condition can impact vancomycin’s effects on kidney function, biodistribution, and elimination. The study revealed a high incidence of AKI before the implementation of the institutional protocol, as well as significant reduction in AKI cases after protocol implementation, as supported by the results.

The institutional protocol demonstrated effectiveness in enhancing the safety of drug therapy, ensuring appropriate therapeutic dosing, and reducing adverse drug events, such as nephrotoxicity, leading to positive patient outcomes.

Future studies are recommended, further investigating and comparing the profiles of patients who received vancomycin therapy and their individual comorbidities.

## Data Availability

The raw data supporting the conclusion of this article will be made available by the authors, without undue reservation.
